# A Haspin-ARHGAP11A axis regulates epithelial morphogenesis through Rho-ROCK dependent modulation of LIMK1-Cofilin

**DOI:** 10.1016/j.isci.2023.108011

**Published:** 2023-09-22

**Authors:** Roberto Quadri, Giuseppe Rotondo, Sarah Sertic, Sara Pozzi, Maria Chiara dell’Oca, Luisa Guerrini, Marco Muzi-Falconi

**Affiliations:** 1 Department of Biosciences, University of Milan, via Celoria 26, 20133 Milan, Italy

**Keywords:** Molecular biology, Cell biology

## Abstract

Throughout mitosis, a plethora of processes must be efficiently concerted to ensure cell proliferation and tissue functionality. The mitotic spindle does not only mediate chromosome segregation, but also defines the axis of cellular division, thus determining tissue morphology. Functional spindle orientation relies on precise actin dynamics, shaped in mitosis by the LIMK1-Cofilin axis. The kinase Haspin acts as a guardian of faithful chromosome segregation that ensures amphitelic chromosome attachment and prevents unscheduled cohesin cleavage. Here, we report an unprecedented role for Haspin in the determination of spindle orientation in mitosis. We show that, during mitosis, Haspin regulates Rho-ROCK activity through ARHGAP11A, a poorly characterized GAP, and that ROCK is in turn responsible for the mitotic activation of LIMK1 and stabilization of the actin cytoskeleton, thus supporting a functional spindle orientation. By exploiting 3D cell cultures, we show that this pathway is pivotal for the establishment of a morphologically functional tissue.

## Highlights

• Rho regulates mitotic spindle orientation through the LIMK1/Cofilin axis
• ARHGAP11A limits Rho activity in M-phase
• Haspin is required to prevent inactivation of Rho by ARHGAP11A
• Loss of Haspin causes dysfunctional spindle orientation and tissue morphogenesis

## Graphical abstract

**Figure:**
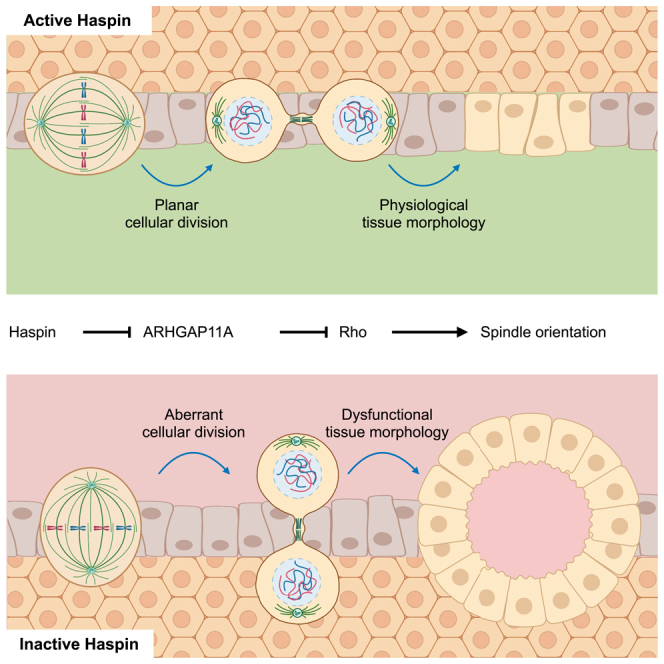

## Introduction

Mitosis is a particularly challenging phase of the cell-cycle as the genetic material must be evenly segregated to daughter cells avoiding the insurgence of aneuploidies. This is ensured thanks to an intricate integration between the mitotic spindle, responsible for mechanically pulling sister chromatids apart, and several checkpoints that delay anaphase onset until mandatory requirements are achieved. For example, centromeric cohesin must be protected from premature cleavage to sustain chromosome biorientation, and anaphase onset must be delayed until all chromosomes are correctly positioned on the metaphase plate. Both these processes rely on the activity of the evolutionarily conserved atypical protein kinase Haspin, which is emerging as a central factor in several types of cancers.^1^ Haspin activity peaks in mitosis, thanks to the presence of an autoinhibitory loop that is folded onto the catalytic domain and is displaced following phosphorylation by mitotic kinases.^2^^,^^3^ Haspin plays a central role in ensuring the establishment of a functional mitotic apparatus, acting at multiple steps.^1^ First, Haspin prevents an unscheduled removal of cohesins from centromeric regions. Upon entry into prometaphase, following binding of the Wapl cohesin dissociation factor to Pds5, cohesin complexes are removed from chromosome arms.^4^^,^^5^^,^^6^^,^^7^^,^^8^ However, to sustain the tension required for chromosome biorientation, centromeric cohesin must remain tightly in place.^9^^,^^10^ At centromeric regions, Haspin physically interacts with Pds5 competing with Wapl itself for the access to Pds5l.^11^^,^^12^ Furthermore, Haspin binds to and phosphorylates Wapl making it unable to interact with Pds5.^13^ The ultimate overall effect is the stabilization of centromeric cohesin. Second, Haspin participates in the response to misattached kinetochores. Haspin phosphorylates threonine 3 of histone H3^14^^,^^15^ (H3-Thr3p) that, among other roles,^16^^,^^17^ promotes the centromeric recruitment of the Chromosomal Passenger Complex (CPC). The CPC consists of Aurora B, Survivin (which binds to H3-Thr3p^18^^,^^19^^,^^20^), Borealin (which binds to Bub1-dependent H2A-T120p^18^^,^^21^^,^^22^^,^^23^) and INCENP. The CPC senses the presence of misattached kinetochores and triggers a cell-cycle delay until a functional metaphase plate is built.^24^ It is thus clear that Haspin oversees multiple critical processes to ensure a correct mitosis. Accordingly, its loss of function leads to anaphase onset in the presence of a dysfunctional metaphase plate^14^ causing lagging chromosomes^25^ and premature chromosome separation.^12^^,^^13^^,^^15^

Sustaining tissue homeostasis and functionality requires proficient chromosome segregation and the proper orientation of cell division. Remarkably, both functions are based on a functional mitotic spindle, as the orientation of the mitotic apparatus defines the future site of cleavage and cell division pattern.^26^ Accordingly, misregulation of spindle orientation results in aberrant multilumen cysts in a 3D cancer colon model.^27^ Spindle orientation is orchestrated by an intricate network of factors,^26^ among which the actin cytoskeleton has a prominent role.^26^^,^^27^^,^^28^^,^^29^^,^^30^ Indeed, in mitosis this structure is heavily reshaped to properly anchor and orient the mitotic spindle and defects in actin cytoskeleton result in spindle misorientation,^31^^,^^32^ eventually leading to aberrant apoptosis or malignant transformation.^30^ Throughout interphase, actin filaments are organized in so-called stress fibers, which are at the bases of cell shape, adhesion and motility.^33^^,^^34^ Upon entry into mitosis, stress fibers and focal contacts are disassembled, and actin is remodeled to form a cortical skeleton anchored to the plasma membrane. Such structure is important to generate the forces required to sustain the mitotic cell rounding.^30^^,^^35^ Actin remodeling is a dynamic process depending mainly on the interplay and balance between actin polymerizing and depolymerizing proteins.^36^^,^^37^ Crucial among these is the actin-depolymerizing factor (ADF)/Cofilin that at the beginning of mitosis stimulates the severance and depolymerization of actin filaments, thus allowing cell roundup.^34^^,^^38^^,^^39^^,^^40^^,^^41^ The newly built cortical actin skeleton is then stabilized via the inhibitory phosphorylation at Cofilin-Ser3 by the LIM Kinases family of proteins, whose main member is LIMK1.^42^^,^^43^^,^^44^^,^^45^ Failure to phosphorylate Cofilin-Ser3 in prometaphase and metaphase impairs the actin cytoskeleton causing defective mitotic spindle assembly and orientation.^35^ Unscheduled triggering of this pathway is prevented because LIMK1 needs to be fully activated through its phosphorylation on Thr508,^46^^,^^47^ which depends upon the Rho family of small GTPases. Depending on the specific cellular process (i.e., focal adhesion, migration, axon outgrowth, metastasis^47^^,^^48^^,^^49^^,^^50^), LIMK1 may be activated either by ROCK1, a kinase effector of Rho, or by PAK1, a Cdc42/Rac1 effector kinase.^51^^,^^52^^,^^53^^,^^54^^,^^55^^,^^56^^,^^57^

In previous works, we showed that budding yeast Haspin paralogues, Alk1 and Alk2^58^ promote the mitotic resolution of polarity clusters and that failure in this process results in persistent cellular polarization, aberrant actin organization and spindle misorientation.^59^^,^^60^^,^^61^^,^^62^ Here, we investigate the contribution of Haspin to actin remodeling and spindle dynamics in mammalian cells. Our results reveal a conserved role for Haspin in the regulation of spindle orientation and chromosome segregation through reshaping the actin cytoskeleton, complementing the current view of Haspin as major player in the fidelity of mitosis. Indeed, we show that Haspin also contributes to directing cellular division and thus shaping tissue homeostasis. Moreover, for the first time, we provide insights into the physiological functions of ARGHAP11A demonstrating that it modulates Rho activity in mitosis, thus being instrumental to a proficient spindle orientation, and thus to a proper positioning of the cell division axis and tissue determination.

## Results

### Haspin is required for actin-mediated spindle orientation

A functional orientation of the mitotic spindle is a strict requirement for tissue organization, as it is instrumental to properly establish asymmetric versus symmetric cell divisions and to position the future site of cleavage.^26^ We have previously shown that Haspin is important, under certain conditions, for proper spindle orientation in budding yeast.^59^^,^^60^^,^^61^

**Figure 1:**
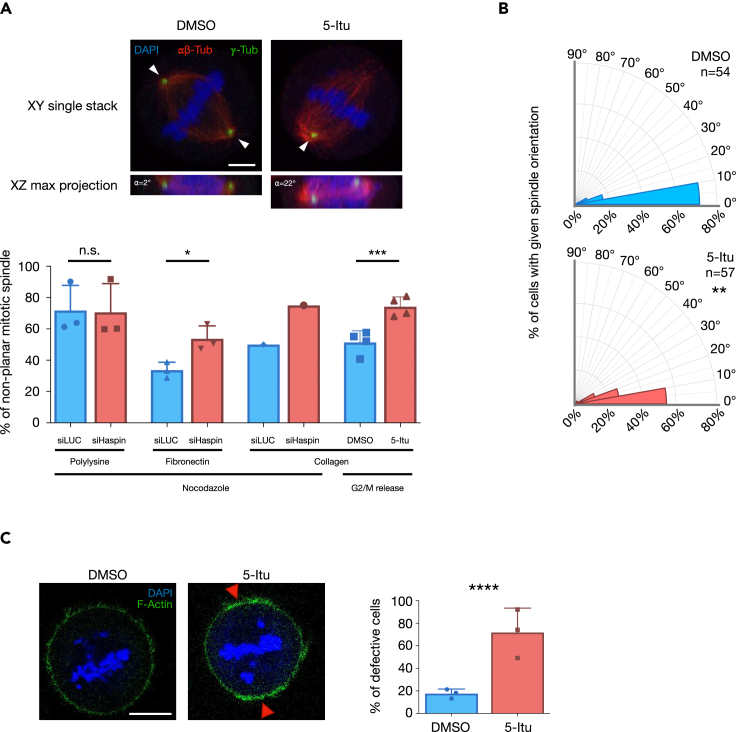
Haspin regulates the actin cytoskeleton to sustain functional spindle orientation (A) HeLa cells were seeded on given substrates and transfected with either control or haspin-targeting siRNAs. Cells were then synchronized in mitosis either by nocodazole or RO-3306 block followed by 1 h release, before being processed to visualize DNA and microtubules. Spindles were counted as misaligned when the two MTOCs (white arrowhead) were on different focal planes. Images were acquired at a confocal microscope, single XY stack and resliced XZ maximum projection are shown; arrowheads point at centrosomes; scale bar: 5μm. The angle between the spindle and the substrate is shown. (B) Cells treated as in A were acquired in confocal microscopy, taking images every 0.5μm, to measure the angle of the spindle compared to the substrate, as shown in Figure S1A. (C) HeLa cells were treated as in panel A, staining the actin cytoskeleton with phalloidin. Arrowheads point at sites of aberrant (increased) actin distribution. Quantification is shown on the right; scale bar: 10μm. Experiments were performed at least three times. Error bars in graphs represent standard deviation, statistical analysis: T-test, significancy: n.s.: not significant; ∗p.value < 0.05; ∗∗p.value < 0.01; ∗∗∗p.value < 0.005; ∗∗∗∗p.value < 0.001.

To investigate whether a similar function is present also in mammalian cells, we seeded HeLa cells on poly-Lysine (a substrate that does not direct a specific spindle orientation), fibronectin or collagen (substrates known to cause a planar orientation of the mitotic spindles),^32^ transfected with control or Haspin-directed siRNAs and then arrested in nocodazole. Cells were then released for 1 h in the absence of nocodazole to allow spindle positioning, and were then fixed and processed to visualize chromatin (DAPI), microtubules (**α**/β-Tubulin) or centrioles (**γ**-Tubulin). The percentage of cells exhibiting non-planar spindles was measured (we defined non-planar spindles when the two centrioles were not visible in the same focal plane at the microscope). As shown in Figure 1A, Haspin inhibition causes an increase in the number of cells with non-planar spindles in conditions where spindle orientation is supposed to be planar (on fibronectin), while it does not impact spindle orientation when cells are grown on poly-lysine. Similar results (Figure 1A) were obtained by growing cells on collagen, or exploiting a different synchronization method (16 h treatment with CDK1/cyclin B1 inhibitor RO-3306^63^), followed by a 1-h release in medium containing 5-Iodotubercidin (a well-established Haspin kinase inhibitor^64^). To better quantify the spindle orientation defect, we measured the spindle angle relative to the substrate (Figure S1A). As shown in Figure 1B, Haspin inhibition causes a misorientation of the mitotic spindles: the fraction of cells having 0–10° angles decreases from 74% in control conditions, to 54% in 5-Itu treated samples.

As mentioned, the orientation of the mitotic spindle is a function of actin cytoskeleton organization.^29^ We thus tested whether loss of Haspin activity impacts on actin cytoskeleton. We plated HeLa cells on fibronectin-coated glass slides and synchronized them at the G2/M transition by treatment with RO-3306. As in previous experiments, 1 h before the release, DMSO or Haspin inhibitor (5-Itu) were added, cells were then washed and released from the G2/M arrest in fresh media containing DMSO or 5-Itu. After 60′ cells were fixed and the actin cytoskeleton was analyzed in metaphase cells (identified by the presence of a metaphase plate) scoring those that exhibited a physiological or altered actin cytoskeleton. While control metaphase cells exhibit a round shape, with even cortical actin distribution (Figure 1C), Haspin inhibition (shown in Figure S1B) causes the accumulation of cells with a defective actin organization encompassing failures in cell roundup and cortical regions with uneven cortical actin distribution, in agreement with the misaligned spindle. Together, these results reveal an evolutionarily conserved role for Haspin in promoting physiological spindle orientation through modulation of the actin cytoskeleton.

### Haspin positively regulates LIMK1-dependent Cofilin-Ser3 phosphorylation

We then committed to the identification of the molecular pathway through which Haspin affects the reorganization of the actin cytoskeleton underlying mitotic round-up, which is under the control of the LIMK1-cofilin axis.^38^^,^^39^^,^^40^^,^^41^^,^^42^^,^^43^^,^^44^^,^^45^ Briefly, LIMK1, triggered through phosphorylation at Thr508, inactivates Cofilin by phosphorylating it on Ser3, preventing unscheduled actin cytoskeleton severance. Inhibition of LIMK1, hence, causes a destabilization of the actin cytoskeleton and aberrant spindle orientation.^35^ Accordingly, in our experimental setup, we observed mitotic spindle misorientation upon treatment with LIMK1 inhibitor BMS4 (Figure S1C), recapitulating what was observed upon loss of Haspin activity.

**Figure 2:**
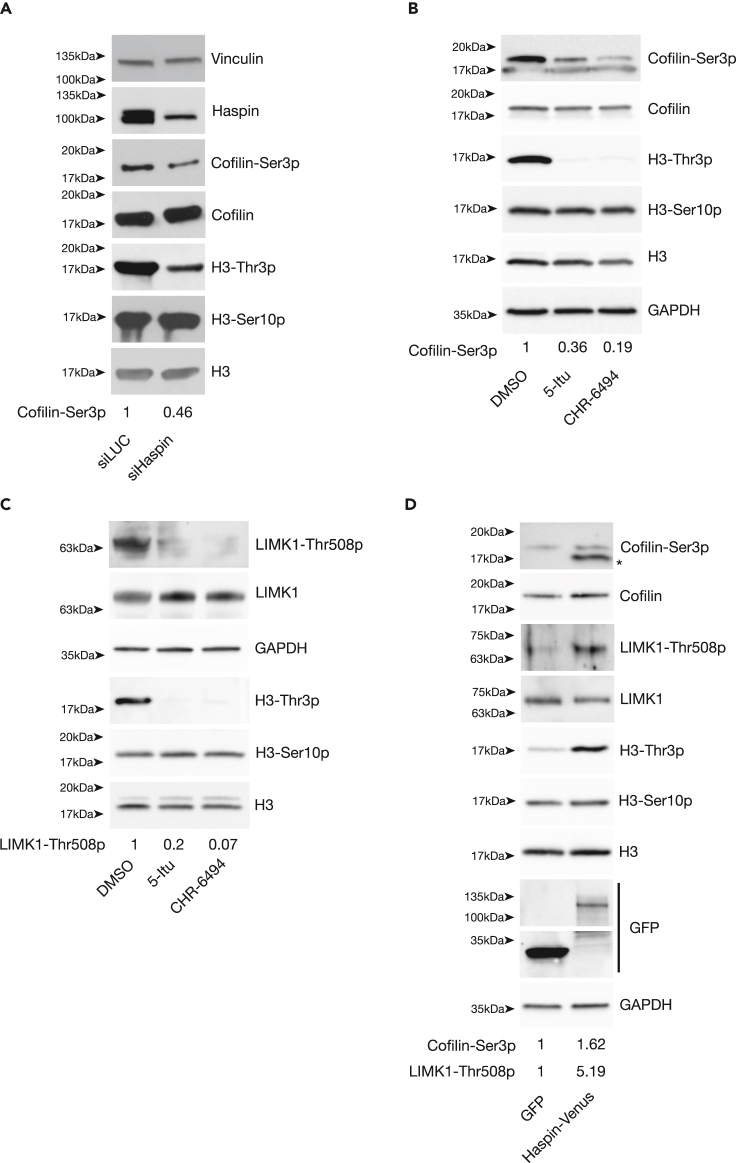
Haspin stimulates LIMK1 activity to inhibit Cofilin Protein levels were analyzed by western blotting with specific antibodies as indicated. HeLa cells were seeded on fibronectin-coated plates, synchronized by nocodazole treatment and: (A) transfected with either Luc- or Haspin-targeting siRNAs (48 h before sample collection); (B and C) inhibited for Haspin by incubation with either 10nM 5-Itu or 50nM CHR-6494 for 1 h before sample collection (see Figure S2A for cell-cycle analyses) or (D) transfected with GFP or Haspin-Venus coding plasmids 24 h before nocodazole treatment (for cell synchronization controls, refer to Figure S2B). For all the experiments, isolation of mitotic cells was achieved by mitotic shake off at the end of nocodazole treatment. Experiments were performed three times (A and C), five times (B) or two times (D). Cofilin-Ser3p/Cofilin (panels A, B, and D) or LIMK1-Thr508p/LIMK1 (panels C and D) ratios are reported.

We then analyzed the impact of Haspin loss on Cofilin in cells arrested in mitosis. As shown in Figure 2A, we observed a consistent reduction in the levels of Cofilin-Ser3p, indicating an increase in cofilin activity upon Haspin silencing. We excluded significant differences in cell-cycle distribution, as H3-Ser10p, a known mitotic mark, was not affected by loss of Haspin. We validated and extended this result by inhibiting Haspin activity either with 5-Itu or exploiting a second independent chemical inhibitor, CHR-6494.^65^ As shown in Figures 2B and S2A, Haspin inhibition led to a decrease in Cofilin-Ser3p (Figures 2B and S2A; remarkably the treatment did not impair cell proliferation; Figure S2B).

Considering the reported role for LIMK1 in Cofilin-Ser3 phosphorylation^42^^,^^43^^,^^44^^,^^45^ and the observed reduction in this PTM upon loss of Haspin activity, we then monitored the abundance of active LIMK1 (LIMK1-Thr508p^46^^,^^47^). As shown in Figure 2C, Haspin inhibition causes a decrease in active LIMK1, consistent with the observed failure in Cofilin inactivation. Finally, we validated these results through Haspin overexpression by transfecting cells with either a GFP or a Haspin-Venus^66^ construct. As shown in Figures 2D and S2C, overexpression of Haspin caused the accumulation of active LIMK1 (LIMK1-Thr508p) and a concomitant reduction of active Cofilin. Noteworthy, Haspin-mediated regulation of Cofilin1 phosphorylation is not exclusive to HeLa cells, as similar results were observed both in HEK-293T or CaCo2 cells (Figures S2D and S2E). These findings suggest that Haspin inhibits cofilin activity through promotion of the activatory phosphorylation of LIMK1.

### The LIMK1-Cofilin system is regulated by Rho-ROCK1 in early mitosis

**Figure 3:**
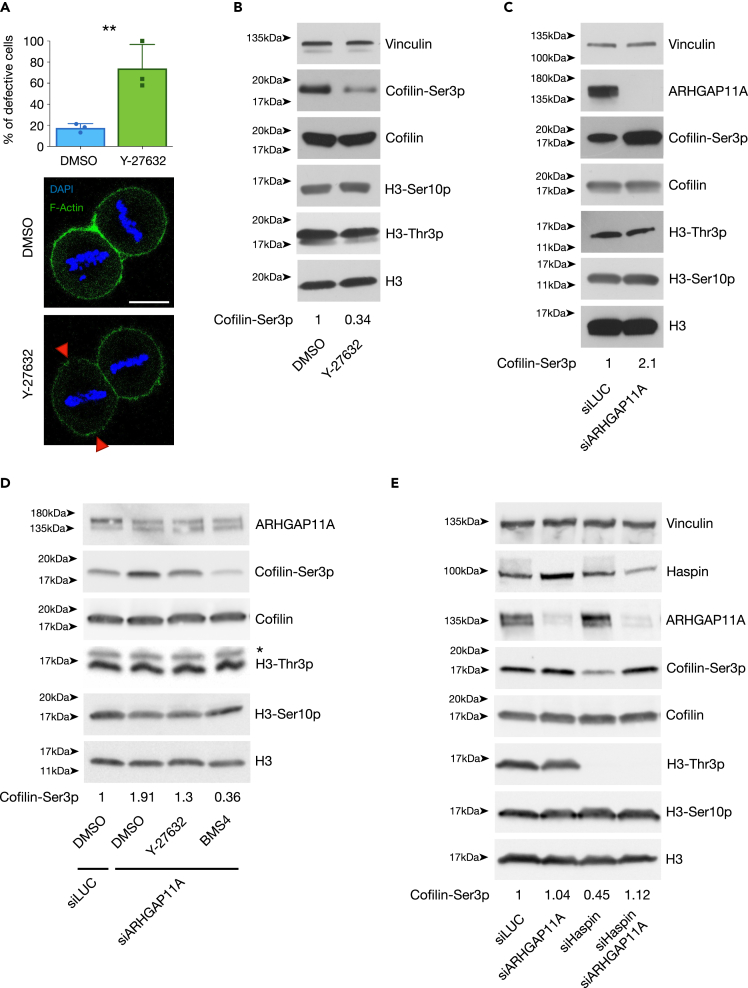
A Haspin-ARHGAP11A-Rho-ROCK axis modulates mitotic Cofilin phosphorylation Protein levels were analyzed by western blotting with specific antibodies as indicated. HeLa cells seeded on fibronectin-coated plates were treated as follows. (A and B) Cells were synchronized at the G2/M transition by RO-3306 treatment and released for 1 h in the absence or presence of 10μM ROCK inhibitor Y-27632. Cells were then processed to visualize actin cytoskeleton (by Alexa Fluor-phalloidin) and quantitate cells with defective actin organization (A; scale bar: 10μm) or monitor the phosphorylation status of Cofilin (B). Arrowheads in A point at sites of aberrant (increased) actin distribution. (C–E) isolation of mitotic cells was achieved by nocodazole treatment followed by mitotic shake off; silencing of given proteins was achieved by transfecting siRNAs 24 h before nocodazole treatment. Chemical inhibition of ROCK (Y-27632) and LIMK1 (BMS4) in D was performed adding the drugs 1 h before sample collection. Experiments were performed three times (A,C,E) or two times (B,D). Error bars in graphs represent standard deviation, statistical analysis: T-test, significancy: n.s.: not significant; ∗p.value < 0.05; ∗∗p.value < 0.01; ∗∗∗p.value < 0.005; ∗∗∗∗p.value < 0.001. Cofilin-Ser3p/Cofilin ratio is reported in panels B–E.

Two main mechanisms oversee and cooperate in the regulation of LIMK1 activity, one orchestrated by Cdc42 and its effector PAK, and one relying on Rho and its effector ROCK. In yeast, we have shown that Haspin is required for the rerouting of Ras-GTP loaded vesicles from a polarized to an isotropic delivery,^59^ this ultimately promotes a redistribution of actin cytoskeleton and polarity factors in a Cdc42-mediated manner.^59^^,^^61^ We thus tested the possible involvement of Cdc42 in the regulation of mitotic LIMK1 activity by performing RNA interference against CDC42. Silencing of Cdc42 in our system did not result in evident changes of the abundance of Cofilin-Ser3p in mitotic HeLa cells (Figure S3A), consistently with previous reports,^67^ and excluding that the phenotypes observed upon loss of Haspin activity might rely on altered Cdc42 signaling. On the other hand, inhibition of the Rho-ROCK pathway by treatment with Y-27632^68^ caused a significant increase in the percentage of cells exhibiting an aberrant actin cytoskeleton (Figure 3A) and a concomitant reduction in phosphorylated (inactive) Cofilin (Figure 3B), depicting Rho-ROCK as the main regulators of this signaling pathway in mitosis.

### Haspin regulates the Rho-ROCK1-LIMK1-cofilin pathway through the modulation of ARHGAP11A phosphorylation in early mitosis

So far, our results position Haspin in the Rho-ROCK-dependent regulation of the LIMK1-Cofilin axis to modulate actin cytoskeleton and spindle orientation but provide no insights as to how Haspin might impact on Rho itself. The activity of GTPases is under control of different classes of proteins that have either activating (GEFs) or inhibiting (GAPs and GDIs) roles; a convenient mechanism by which such regulators are triggered or inactivated is through phosphorylation. We thus speculated that Haspin might impinge on Rho-GTP/Rho-GDP cycle modulation by phosphorylating one of its GEFs, GAPs or GDIs. Interestingly, Haspin regulates the phosphorylation of ARHGAP11A,^69^ a Rho GAP with a role in confining mitotic RhoA activity at the equatorial axis.^70^

We then assessed whether ARHGAP11A acts as a negative regulator of the Rho-ROCK pathway in mitotic cells. We depleted ARHGAP11A by RNA interference, synchronized cells in prometaphase by nocodazole treatment and monitored Cofilin-Ser3p levels. As shown in Figure 3C, loss of ARHGAP11A, while not affecting Haspin activity (H3-Thr3p) or the amount of mitotic cells (H3-Ser10p), caused a marked increase in phosphorylated Cofilin, consistent with an hyperactivation of the Rho-ROCK-LIMK1 pathway. Corroborating this result, inhibition of ROCK or LIMK1 abrogated the hyperphosphorylation of Cofilin-Ser3p in ARHGAP11A-silenced cells (Figure 3D). If Haspin-dependent phosphorylation is critical to control the activity of ARHGAP11A and, consequently, the LIMK1-Cofilin axis, removal of ARHGAP11A should rescue the effects of Haspin inhibition. Indeed, as shown in Figure 3E, while Haspin silencing causes a decrease in inactive Cofilin (pCofilin), simultaneous silencing of ARHGAP11A prevents this reduction in Cofilin-Ser3p levels, confirming that Haspin regulates Cofilin phosphorylation through the ARHGAP11A-Rho-ROCK-LIMK1 axis. Remarkably, loss of Haspin resulted in increased ARHGAP11A protein levels, supporting a role for Haspin-regulated ARHGAP11A phosphorylation in promoting mitotic degradation of this GAP and the consequent cofilin inactivation.

### Haspin activity is required for proper cell growth patterns

**Figure 4:**
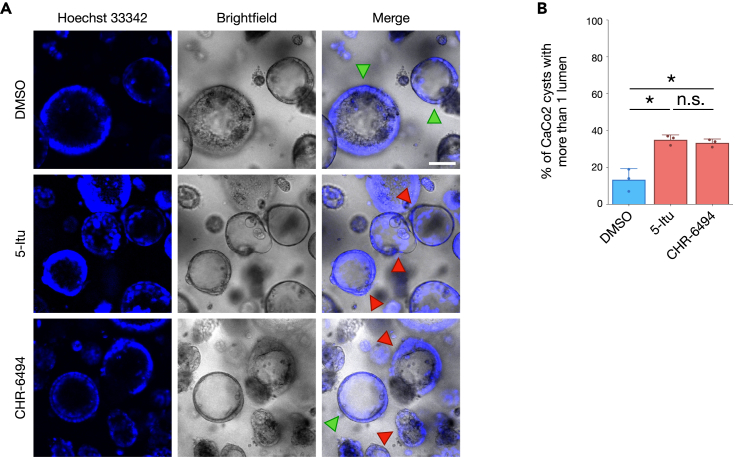
Impairments in Haspin activity cause tissue alterations and development issues (A) 3D Caco2 cultures grown in a Matrigel-collagen matrix were incubated for 12 days in the presence of either DMSO, 10nM 5-Itu or 50nM CHR-6494, adding 0.1 mg/ml cholera toxin for the last 12 h, before being stained with Hoechst 33342 and analyzed by fluorescent and brightfield microscopy. Panel A shows representative examples, green and red arrowheads point to single lumen or multilumen cysts, respectively; scale bar: 100μm. (B) Plot shows the percentage of multilumen cysts in control or Haspin-inhibited conditions. Experiment was performed two times. Error bars in graphs represent standard deviation, statistical analysis: T-test, significancy: n.s.: not significant; ∗p.value < 0.05; ∗∗p.value < 0.01; ∗∗∗p.value < 0.005; ∗∗∗∗p.value < 0.001.

Having proved the involvement of Haspin kinase in the control of spindle orientation, we tested the physiological relevance of this pathway. CaCo2 cells can easily be cultured in 3D to form cysts embedded in Matrigel, and, if spindle orientation is proficient, they will generate a hollow sphere with a single lumen.^27^ On the other hand, impairment in spindle orientation will cause aberrant cell division axis, generating cysts with more than one lumen.^27^ We exploited this experimental setup to verify whether loss of Haspin activity might impact on epithelial cell organization and, in principle, tissue morphogenesis (remarkably, Haspin inhibition in these conditions did not significantly alter cell proliferation as measured by MTS assay, Figure S4A). As shown in Figures 4A and 4B, after 12 days of growth Haspin inhibition through either 5-Itu or CHR-6494 caused a significant increase in cysts with more than one lumen (and, accordingly, a concomitant reduction of cyst circularity, particularly evident in cysts with multiple lumen, Figure S4B), implying a role for the Haspin-ARHGAP11A-RHO-ROCK axis in tissue morphogenesis. Similarly to 2D cell cultures, this phenotype is likely ascribable to a defective orientation of the mitotic spindle, as Haspin inhibition caused an increase in mitotic cells with misoriented mitotic chromosomes (Figures S4C and S4D).

## Discussion

The atypical kinase Haspin has been reported to be central in chromosome segregation by acting at multiple levels. First, it phosphorylates H3-Thr3^13^^,^^15^ at centromeric regions, a PTM that is read by Survivin,^18^^,^^19^^,^^20^ a subunit of the Chromosomal Passenger Complex (CPC). This, along with the phosphorylation of H2A-Thr120 by Bub1,^18^^,^^21^^,^^22^^,^^23^ mediates the buildup of a centrosomal pool of the CPC, which in turns detects misattached kinetochores and triggers a cell-cycle delay preventing anaphase until a functional metaphase plate is achieved.^24^ Second, Haspin prevents the unscheduled removal of centromeric cohesin by both competing with Wapl for a physical interaction with Pds5,^11^^,^^12^ and by directly phosphorylating Wapl lowering its affinity for Pds5.^13^ Haspin loss thus results in anaphase onset even in the presence of a dysfunctional metaphase plate^14^ leading to lagging chromosomes^25^ and premature chromosome separation events.^12^^,^^13^^,^^15^

In most eukaryotes, the mitotic spindle defines the axis of cell division and thus its orientation is tightly regulated to properly shape tissue morphology. A major determinant of spindle orientation is the actin cytoskeleton, which undergoes extensive reshaping throughout mitosis to allow mitotic roundup and sustain the forces necessary for cell division. Work in budding yeast has shown that Haspin promotes a redistribution of polarity factors and a reshaping of the actin cytoskeleton, ensuring a functional orientation of the mitotic spindle.^59^^,^^60^^,^^61^ In this work, we report that Haspin controls proper spindle orientation in mammalian cells, and describe an unprecedented axis linking Haspin to Cofilin and actin dynamics, determining a new function for RhoA GAP ARHGAP11A. Remarkably, in a previous *in silico* analysis, ARHGAP11A was linked to mitotic nuclear division, cellular polarity, microtubule-based movements and chromosome organization, supporting our findings.^71^ We show here that, in mitosis, Haspin modulates the activity of the Rho-ROCK pathway by lowering ARHGAP11A protein levels. Rho in turn regulates the accumulation of active, Thr508-phosphorylated LIMK1, as already reported in meiosis.^72^ Finally, phosphorylated LIMK1 inactivates Cofilin, thus stabilizing the mitotic actin cytoskeleton and allowing proper spindle orientation. It is intriguing that Haspin kinase controls spindle orientation in two systems as different as budding yeast^59^^,^^61^ and human cells, where the establishment of the axis of cell division is completely different. In *Saccharomyces cerevisiae*, the decision regarding where the cell division plane will be built is taken in G1 and it is the mitotic spindle that needs to find the proper orientation. In human cells, the axis of cell division is determined in M following the assembly and orientation of the mitotic spindle. In this context, the misorientation of the spindle upon loss of Haspin activity causes an altered cell division pattern that comes with apparently limited impact on the single cell fitness (though it might severely affect cells that must undergo asymmetric division), but has a dramatic morphological effect when it comes to tissue.

**Figure 5:**
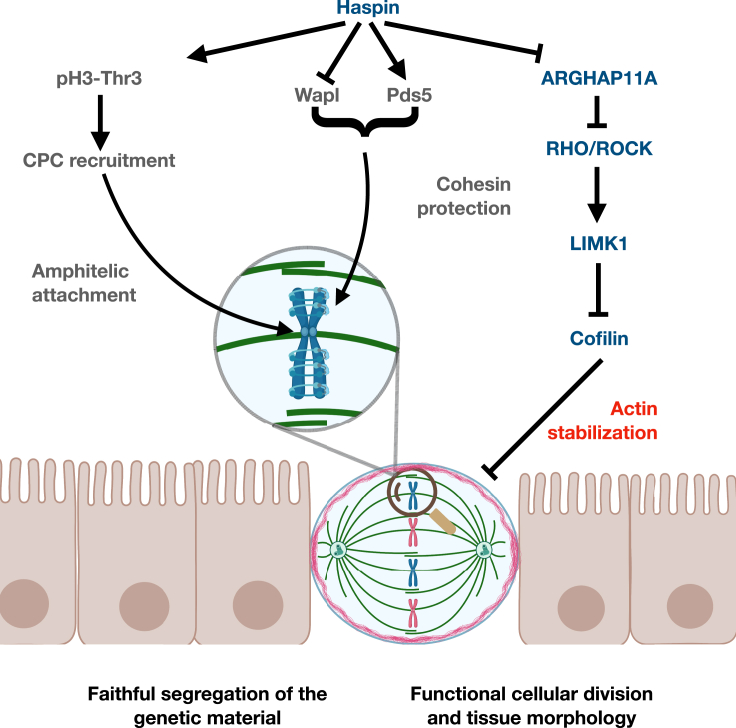
Haspin activity is required for spindle orientation and a successful mitosis In mitosis, Haspin negatively regulates ARGHAP11A, preventing its excessive accumulation (blue text). This results in a buildup of Rho activity that, through the ROCK-LIMK1 axis promotes the inhibitory phosphorylation of Cofilin, stabilizing the cortical actin cytoskeleton and supporting a functional orientation of the mitotic spindle. Together with its established in monitoring amphitelic attachment of the chromosomes and in preventing unscheduled cohesin cleavage (gray text), these findings depict Haspin as a central player in mammalian cell mitosis to orchestrate not only an even distribution of the genetic material between daughters, but also maintaining a functional tissue organization.

These findings are particularly interesting, as they extend the range of mitotic events orchestrated by Haspin from those strictly related to sister chromatids dynamics (cohesin stabilization and amphitelic attachment) to a much wider guardian of the whole segregation process. Failures in Haspin activity thus come with a complex impact on the M-phase, leading to cells susceptible to fail in several of the tight requirements to mitosis and thus prone to genome instability (due to unscheduled cohesin cleavage and errors in the buildup of a metaphase plate) or tissue outgrowth and cell migration (due to misoriented mitotic spindles and cell divisions), being overall primed for malignant transformation (Figure 5). Spindle orientation is a key determinant of the cell division axis and hence tissue homeostasis, and defects in its alignment are linked to carcinogenesis.^73^ Overall, our results, along with the reported role played by Haspin on cohesin dynamics and spindle assembly checkpoint, are instrumental for the comprehension of the advantage provided to malignant cells by the observed LOH of Haspin chromosome arm frequently observed in tumors.^1^

### Limitations of the study

Our work described a novel axis regulating spindle orientation in epithelial cells, highlighting an unprecedented involvement of the newly described Haspin-ARHGAP11A-Rho axis in such process. Beside the conceptual advance of this discovery in the elucidation of how epithelial tissues are shaped, some aspects remain to be addressed. How is this role of the Haspin kinase regulated and how does it integrate with previously described Haspin functions? What are the spatiotemporal dynamics of ARHGAP11A-mediated regulation of Rho? Does the Haspin-ARHGAP11A axis regulate other functions of Rho? All these questions remain open and will need further experimental investigation to be addressed.

## STAR★Methods

### Key resources table

**Table:** 

REAGENT or RESOURCE	SOURCE	IDENTIFIER
**Antibodies**		

Rabbit anti-GFP	Clontech	632677
Rabbit anti-histone H3	Abcam	Ab1791; RRID: AB_302613
Mouse anti-Vinculin	Sigma-Aldrich	V9131; RRID: AB_477629
Rabbit anti-Haspin	Abcam	Ab226222; RRID: AB_3065174
Rabbit anti-phosphorylated H3-Thr3	Upstate	05–-746; RRID: AB_10863137
Mouse anti-phosphorylated H3-Ser10	Abcam	Ab14955; RRID: AB_443110
Rabbit anti-phosphorylated Cofilin-Ser3	Cell Signaling	3313; RRID: AB_2080597
Rabbit anti-Cofilin	Cell Signaling	5175; RRID: AB_10622000
Rabbit anti-phosphorylated LIMK1-Thr508	Abcam	Ab131341; RRID: AB_11159530
Rabbit anti-LIMK1	Cell Signaling	3842; RRID: AB_2281332
Rabbit anti-ARHGAP11A	Biorbyt	Orb125443; RRID: AB_3065175
Rabbit anti-CDC42	Cusabio	PA005008DA01HU; RRID: AB_3065176
Rabbit anti-**γ**Tubulin	Sigma-Aldrich	T5192; RRID: AB_261690
Rat anti-**α**Tubulin	Abcam	Ab6160; RRID: AB_305328
AlexaFluor488 goat anti-rabbit	ThermoFisher Scientific	A11034; RRID: AB_2576217
AlexaFluor594 goat anti-rat	ThermoFisher Scientific	A11007; RRID: AB_10561522
AlexaFluor488 conjugated phalloidin	Immunological Sciences	PP-10052

**Bacterial and virus strains**		

DH5α competent cells	ThermoFisher Scientific	18,265,017

**Chemicals, peptides, and recombinant proteins**		

Penicillin-Streptomycin	EuroClone	ECB3001
Formaldehyde	Sigma-Aldrich	P6148
RNAse A	Sigma-Aldrich	R6513
Hoechst 33342	BioRad	639
ProlongGold with DAPI	ThermoFisher Scientific	P36931
Immersion oil type F	ThermoFisher Scientific	11,944,399
Propidium Iodide	Sigma-Aldrich	P-4864
Poly-L-lysine	Sigma-Aldrich	P-4707
Fibronectin	Sigma-Aldrich	F0895
Matrigel	Corning	CLS354234-1EA
Collagen	Merck	CLS354236
RO-3306	Sigma-Aldrich	SML0569
Nocodazole	USBio	N3000
5-Iodotubercidine	Sigma-Aldrich	I1000
CHR-6494	Sigma-Aldrich	SML0648
BMS4	Axon MedChem	1949
Y-27632	Merck	688092

**Critical commercial assays**		

RNAiMAX transfection reagent	Thermo Fisher Scientific	13778075
Lipofectamine 3000 transfection reagent	Thermo Fisher Scientific	L3000008
Clarity western ECL substrate	BioRad	170-5061
Mini-Protrean TGX 4-20 50μl	BioRad	4568094
CellTiter 96® AQueous One Solution Cell Proliferation Assay	Promega	G3580

**Experimental models: Cell lines**		

HeLa, cervical adenocarcinoma, 31 year old woman	N.A.	N.A.
CaCo2, colorectal adenocarcinoma, 72 year old man	N.A.	N.A.
HEK-293T, embryo kidney tissue, human fetus	N.A.	N.A.

**Oligonucleotides**		

siLUC	CGUACGCGGAAUACUUCG	
siHaspin	GAUCACUAUAAUUCAACCA	
siCDC42	CGAUGGUGCUGUUGGUAAATT	
siARHGAP11A	AGAAGUAGAUGCAAAUGAATT	

**Recombinant DNA**		

pEGFP-C3	N.A.	
Haspin-Venus	Balzano et al.^66^	

**Software and algorithms**		

ImageLab	BioRad	https://www.bio-rad.com
FIJI	NIH	https://ImageJ.nih.gov
R	R core team	https://www.R-project.org/
Graph-pad Prism 7	Graphpad.com	https://www.graphpad.com

**Other**		

Widefield microscope	Leica	DMRA2
Laser scanning confocal microscope	Leica	SPE
Spinning disk microscope	Nikon	CSU-W1
Plate reader	Perkin Elmer	Ensight

### Resource availability

#### Lead contact

Further information and requests for resources and reagents should be directed to and will be fulfilled by the lead contact, Marco Muzi-Falconi (marco.muzifalconi@unimi.it).

#### Materials availability

This study did not generate new unique reagents.

### Experimental model and study participant details

HeLa, HEK293T and CaCo2 cells were grown in DMEM supplemented with 10% FBS and Penicillin/Streptomycin. To obtain 3D cultures of CaCo2, 400μl of cells (1∗10ˆ6 cells/ml) were mixed with 400μl of Matrigel and 200μl of Collagen I. 200μl of the mix were then plated on the bottom of an imaging dish and solidified by incubation at 37°C before medium and drug addition.

### Method details

#### Cell synchronization

Mitotic cells were obtained either by synchronization at the G2/M transition by treatment with CDK1 inhibitor RO-3306 (10μM) followed by 1 hour release, or by synchronization at prometaphase by a 16 hours incubation in the presence of 100nM Nocodazole. In both cases, mitotic cells were obtained by mitotic shake off.

#### Immunofluorescence and fluorescence microscopy

Fluorescence microscopy was exploited to assess spindle orientation and actin organization in mitotic cells; in both cases, cells were fixed with formaldehyde and then processed as follows. For spindle orientation analyses, cells were stained for **γ**-tubulin and **α**-tubulin; while actin staining was performed by Alexafluor 488-conjugated phalloidin. Slides were mounted with a DAPI-containing mounting, before being analyzed by widefield (Leica DMRA2) or confocal microscopy (Leica SP2 or Nikon CSU-W1). Images were analyzed with FIJI.^74^

#### Cell transfection

Cells were transfected 48 hours before sample collection; plasmids for overexpression were transfected using Lipofectamine 3000 following provider’s manual; siRNAs were transfected using RNAiMAX following provider’s instruction.

#### Western blotting

Cells were lysed in Laemmli buffer, samples were then boiled for 10 minutes and sonicated. After SDS-PAGE and transfer to nitrocellulose membrane, filters were blocked with 5% milk in PBS TWEEN-20 0.1% (PBST) and then incubated with given primary antibody. Filters were washed 3 times in PBST, incubated 1 hour at RT with secondary HRP-conjugated antibody, washed for further 3 times. ChemidocTouch (BIORAD) was used to acquire images.

#### FACS analyses

Samples were prepared for flow cytometry as previously described.^75^ Briefly, cells were detached through trypsinization, washed in PBS and fixed in 70% ice-cold ethanol. Samples were then washed with PBS/BSA and stained with 20 ug/mL propidium iodide, 10 ug/mL RNase A at room temperature for 30 min. FACS analyses were performed on a BD FACScan or BD Accuri and quantified with Cell Quest software (BD Bioscience).

#### Cell proliferation measurement

To evaluate cellular proliferation in 2D cultures, cells were fixed with 3.7% formaldehyde at given time points and then stained with crystal violet. After washout of the dye, cells were lysed with 1% SDS and absorbance at 595 nm measured with a Perkin Elmer Ensight. CaCo2 proliferation in cysts was performed incubating cells embedded in Matrigel with MTS assay for 2 hours and then reading absorbance at 490 nm.

### Quantification and statistical analyses

Statistical significancy was evaluated by T-test, significancy: n.s.: not significant; ∗ p.value <0.05; ∗∗ p.value <0.01; ∗∗∗ p.value <0.005; ∗∗∗∗ p.value <0.001. Further details regarding number of experimental repeats or number of analyzed cells and graph description, are reported in figure legends.

## Data Availability

• All data reported in this paper will be shared by the lead contact upon request. • This paper does not report original code. • Any additional information required to reanalyze the data reported in this paper is available from the lead contact upon request.
