# Supplementary material for: A Haspin-ARHGAP11A axis regulates epithelial morphogenesis through Rho-ROCK dependent modulation of LIMK1-Cofilin
Source: iScience. 2023 Sep 22;26(10):108011. doi: 10.1016/j.isci.2023.108011 (PMC10570125; doi:10.1016/j.isci.2023.108011)
Supplement: Document S1. Figures S1–S4 [file mmc1.pdf]

iScience, Volume *26*

Supplemental information

A Haspin-ARHGAP11A axis regulates epithelial morphogenesis through Rho-ROCK dependent modulation of LIMK1-Cofilin

Roberto Quadri, Giuseppe Rotondo, Sarah Sertic, Sara Pozzi, Maria Chiara dell'Oca, Luisa Guerrini, and Marco Muzi-Falconi

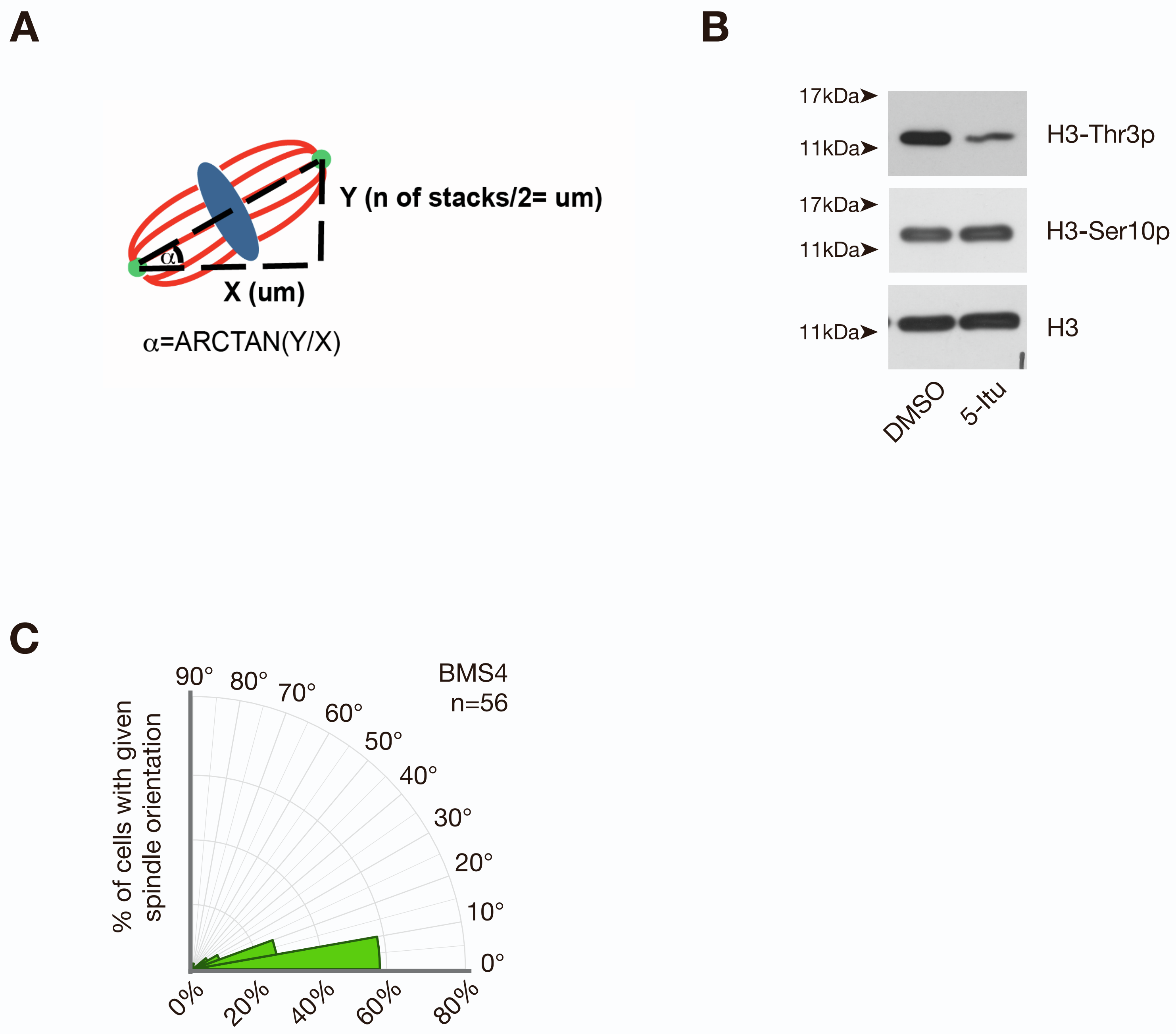

Figure S1: 5-Itu effectively inhibits Haspin in our experimental conditions and the resulting phenotypes are recapitulated by LIMK1 inhibition, related to Figure 1. A) schematic representation of the approach used to determine spindle angle. B) Haspin inhibition control for experiment in Figure 1. C) Spindle angles were measured in cells incubated with LIMK1 inhibitor BMS4 and then processed as in Figure 1B.

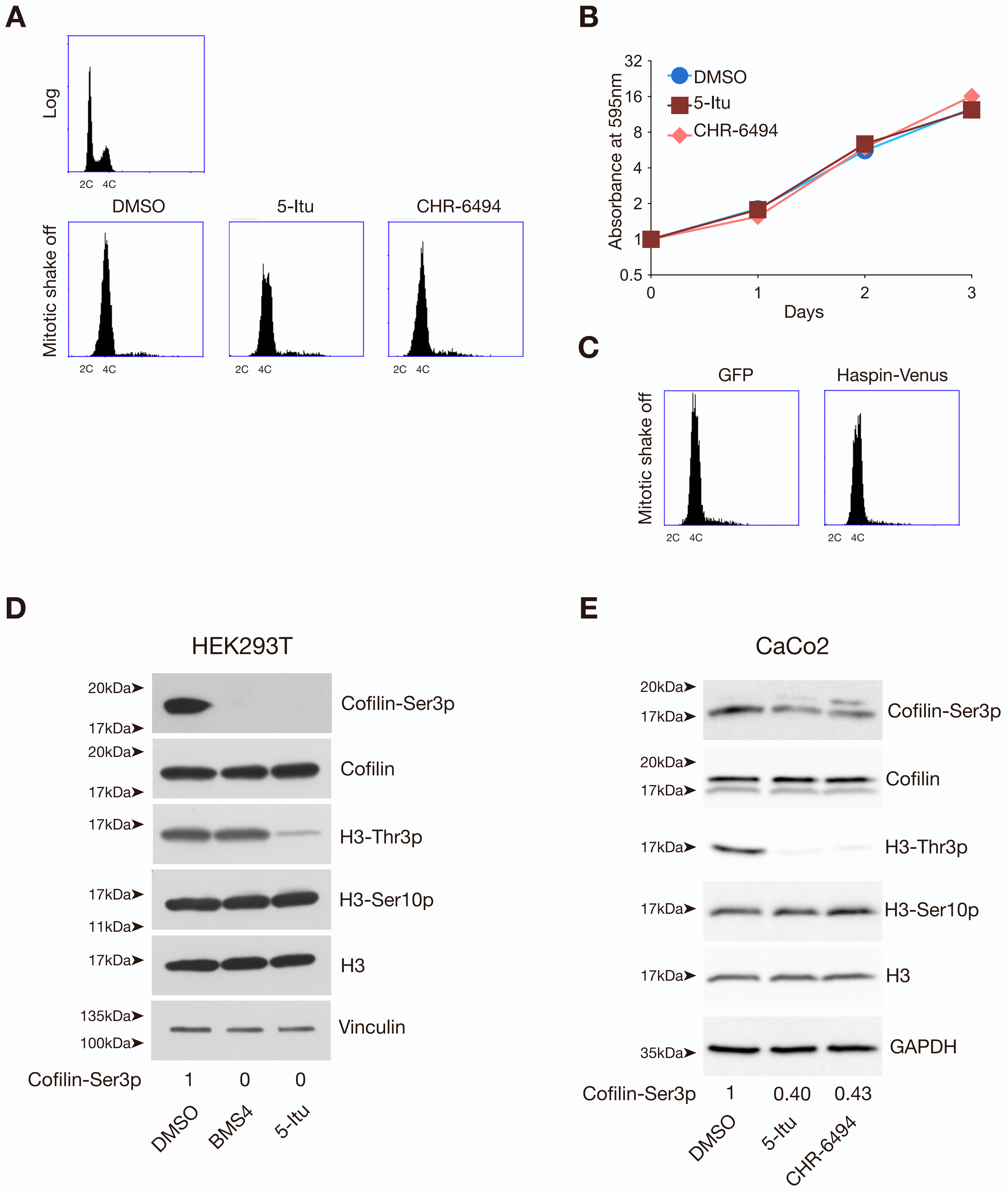

Figure S2: Haspin effect on Cofilin phosphorylation does not depend on altered cell proliferation and can be recapitulated in several cell lines, related to Figure 2. A) Cell cycle analyses by flow cytometry of experiments in Figure 2B; a sample of log cells is provided for reference. (B) HeLa cell proliferation in the presence of DMSO, 10nM 5-Itu or 50nM CHR-6494 was evaluated by crystal violet staining at given time points. C) Cell cycle analyses by flow cytometry of experiment in Figure 2Ds. HEK293T (D) or CaCo2 (E) cells were grown on fibronectin, arrested in nocodazole and incubated for 1 further hour in the presence of nocodazole and DMSO or Haspin inhibitors. Mitotic cells were then analyzed for proteins of interest by western blot. Cofilin-Ser3p/Cofilin ratio is reported.

A

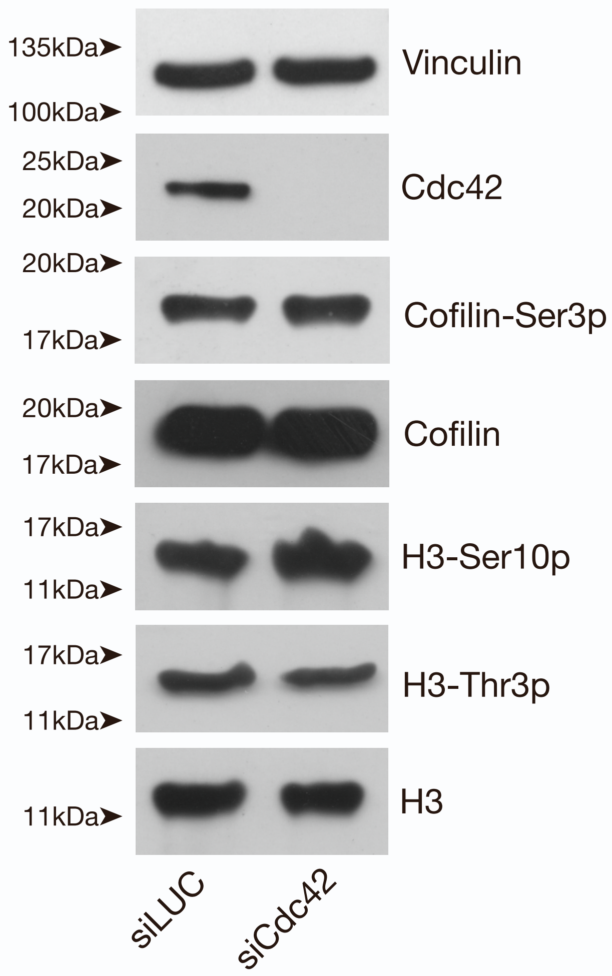

Figure S3: CDC42 is dispensable for Cofilin phosphorylation in mitosis, related to Figure 3. A) Cells were seeded on fibronectin-coated plates, silenced for CDC42 or Luciferase and synchronized in mitosis with Nocodazole. Mitotic cells were obtained by mitotic shake off and the abundance of given proteins was evaluated.

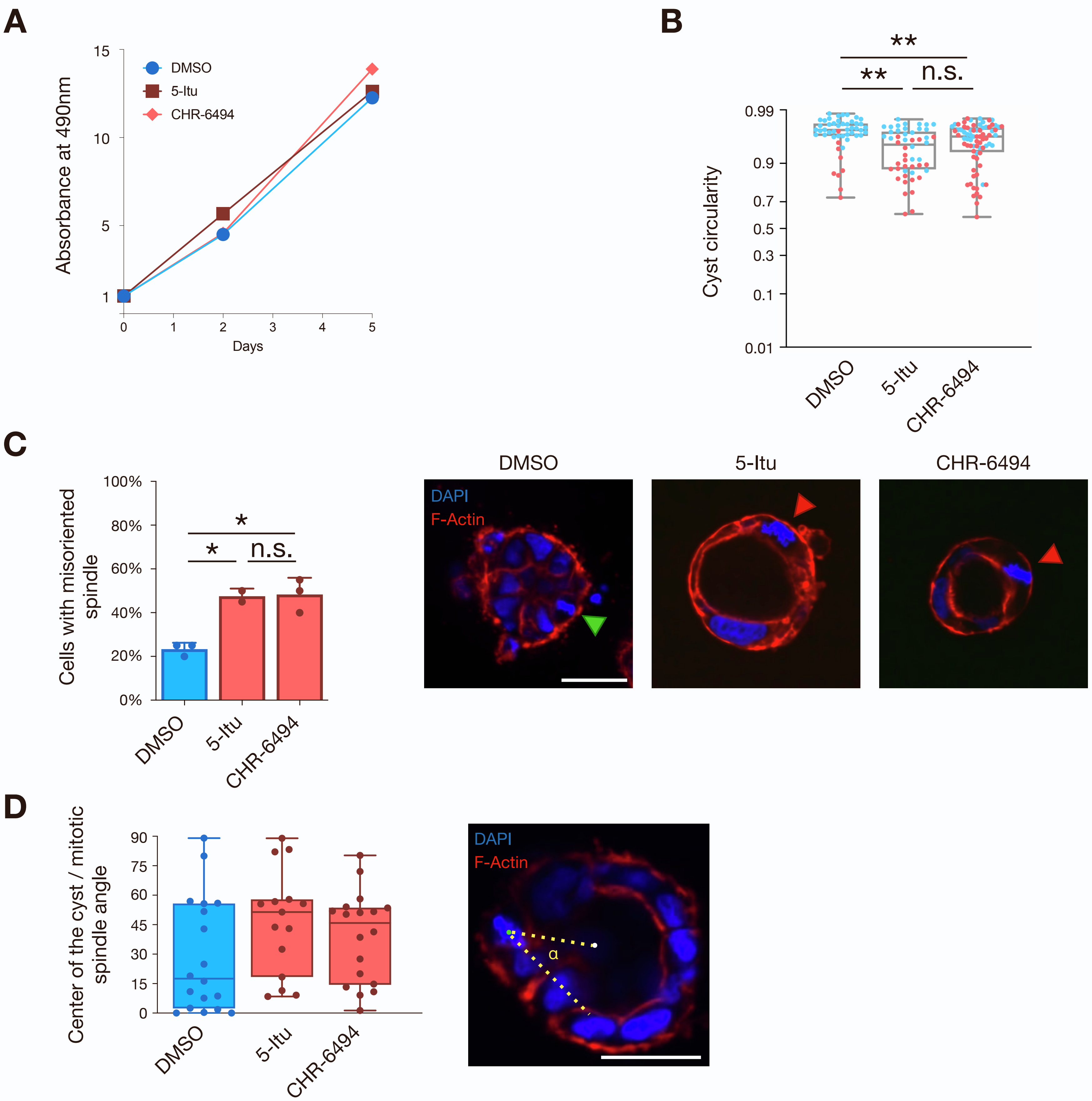

Figure S4: Haspin inhibition results in altered mitotic spindle orientation in 3D CaCo2 cultures, related to Figure 4. A) CaCo2 cells were seeded as described in Figure 4 and then incubated in the presence of DMSO, 10nM 5-Itu or 50nM CHR-6494. At given time points, cell proliferation was evaluated by MTS assay, values are normalized on time 0. B) Cyst circularity was measured on 50 cysts from two independent experiments from Figure 4; boxes represent 50% of data points, line represent mean value, whiskers correspond to minimum and maximum values. Blue and red dots correspond to single or multi lumen cysts, respectively. C) Cells were treated as in Figure 4 and, after 6 days, fixed and stained for DNA (DAPI) and actin (phalloidin) visualization. Graph shows the percentage of mitotic cells within a cyst exhibiting an aberrant spindle orientation as evaluated by eye; 20 cells were counted per experiment. Green and red arrowheads point to properly or aberrantly oriented mitotic chromosomes, respectively; scale bar: 30µm. Statistical analysis: T-test, significancy: n.s.: not significant; * p.value <0.05; ** p.value <0.01; *** p.value <0.005; **** p.value <0.001. D) The angle formed by the mitotic spindle and the center of the cysts was measured as shown (white dot: geometric center of the cyst; green dot: center of the mitotic spindle). Graph shows the median angle value; boxes include 50% of observations, whiskers correspond to minimum and maximum values. Scale bar: 30µm.
